# Who Learns More? Cultural Differences in Implicit Sequence Learning

**DOI:** 10.1371/journal.pone.0071625

**Published:** 2013-08-07

**Authors:** Qiufang Fu, Zoltan Dienes, Junchen Shang, Xiaolan Fu

**Affiliations:** 1 State Key Laboratory of Brain and Cognitive Science, Institute of Psychology, Chinese Academy of Sciences, Beijing, China; 2 Sackler Centre for Consciousness Science and School of Psychology, University of Sussex, Brighton, United Kingdom; University of Tokyo, Japan

## Abstract

**Background:**

It is well documented that East Asians differ from Westerners in conscious perception and attention. However, few studies have explored cultural differences in unconscious processes such as implicit learning.

**Methodology/Principal Findings:**

The global-local Navon letters were adopted in the serial reaction time (SRT) task, during which Chinese and British participants were instructed to respond to global or local letters, to investigate whether culture influences what people acquire in implicit sequence learning. Our results showed that from the beginning British expressed a greater local bias in perception than Chinese, confirming a cultural difference in perception. Further, over extended exposure, the Chinese learned the target regularity better than the British when the targets were global, indicating a global advantage for Chinese in implicit learning. Moreover, Chinese participants acquired greater unconscious knowledge of an irrelevant regularity than British participants, indicating that the Chinese were more sensitive to contextual regularities than the British.

**Conclusions/Significance:**

The results suggest that cultural biases can profoundly influence both what people consciously perceive and unconsciously learn.

## Introduction

It is well documented that East Asians differ from Westerners in conscious perception and attention [1]–[5]. Generally, East Asians are more sensitive to context and relations among objects or events and hence view the world on a global scale, i.e., have a holistic cognitive style, whereas Westerners are more focused on focal objects and hence view the world on a local scale, i.e., have an analytic cognitive style. For example, when describing animated underwater vignettes, Japanese usually first referred to the background (e.g., “it looked like a pool”), while Americans usually first referred to the focal object (e.g., “there was a big fish”) [1], [3]. Or, for example, when clustering three nouns (e.g., chicken, cow and grass), Chinese tended to group on the basis of thematic relationship (e.g., cow and grass), whereas Americans tended to group on the basis of common category membership (e.g., chicken and cow) [6].

Although a number of studies have investigated cultural differences in conscious perception and attention, few studies have explored cultural differences in unconscious processes such as implicit learning. Implicit learning is the capacity to pick up information about complex stimulus displays largely without awareness of either the process or the products of learning [7]–[9]. The serial reaction time (SRT) task is one of the most widely used tasks in implicit learning [8], [10]–[17]. In a typical SRT task, a stimulus appears at one of several locations on a computer screen and participants are told to press the corresponding key according to the stimulus location shown on the screen. Unbeknownst to them, the order of the stimuli follows a repeating or structured sequence. People are faster to respond when the sequence is structured similarly to the training phase rather than when the sequence is switched from the training phase, indicating learning of the sequential structure. Moreover, such learning occurs even when people deny that there was a sequence, cannot freely report it, or cannot control its generation, indicating the knowledge is (largely) unconscious [17]–[24].

Implicit learning has been seen as an automatic consequence of selective attention or task relevance [25]–[28]. A minimal amount of attention to relevant task features is needed for implicit learning to occur [9], [29]–[31]. As East Asians and Westerns tend to pay attention differentially at global and local scales, they may implicitly acquire different knowledge, especially when the stimuli include both global and local information.

Navon's global-local task [32] has been widely used as an objective measure of attentional breadth (see [33]; for a review, [34]). When shown hierarchical geometric figures in which a global letter is made out of smaller local letters, people preferentially process at global or local scales dependent on context. For example, by manipulating interdependence and independence primes, [35] found that independence- but not interdependence-primed participants were quicker in identifying local rather than global letters. [36] (see also [37]) replicated the results with Chinese participants and found a global precedence in the interdependence priming condition but a local precedence in the independence priming. Various factors affect the global-local bias for perception of these figures, such as the number of local letters that make up a global letter, exposure duration, and clinical condition [38]–[40], but cultural differences have been surprisingly little investigated. People from a remote interdependent culture, Himba of northern Namibia, showed more pronounced local bias than that has been observed in any other normal population [41]. Given the cultural biases of Asians for interdependence and Westerners for independence, there should be Western-Asian cultural preferences for the scale of attention in Navon letters.

Indeed, [42] investigated whether there were cultural differences in the acquiring unconscious knowledge in implicit learning using Navon letters. They adopted Navon letter strings in the artificial grammar learning (AGL) task, a widely used task in implicit learning. In the training phase, the sequence of letters followed one grammar at the global level and a different grammar at the local level. [42] found that when not specifically instructed to attend at either level, Japanese participants expressed a striking advantage in the acquisition of unconscious knowledge at the global level while UK participants learned unconscious knowledge similarly at both global and local levels. This study provided the first evidence that unconscious knowledge reflects the global-local preferences of different cultures. [42] also found that when participants were instructed to attend to a global or local level, they could learn the corresponding grammar with facility, and no differences between the cultures were then detected.

However, although [42] showed that the cultures differed in attentional habits in ways that produced differences in unconscious knowledge, they left open whether the cultures differed in attentional skills in ways that produced differences in unconscious knowledge. There is evidence for differences in attentional skills in terms of conscious perception. For example, [43] found that when subjects were asked to copy a line drawn in a square onto a different sized square, Americans were more accurate than Japanese when instructed to draw the absolute length of the line independent of the size of the square but Japanese were more accurate than Americans when instructed to draw the same length of line relative to the square. That is, even when instructed to adopt a global or local style, different cultures were still somewhat bound by their habits. It remains open to what extent people can control their attention sufficiently to eradicate cross-cultural differences in implicit learning - or if small cultural differences in attention even when people are instructed to attend a certain way still translate into cultural differences in implicit learning. The current study will address the issue of whether there can be cultural differences in implicit learning even when the cultures are instructed as to which level to attend.

The finding of cultural influences on artificial grammar learning also raises the question of whether such influences occur on other types of implicit learning. Although the AGL and SRT are two widely used tasks in implicit learning, there is little correlation in performance between them [44]. [45] classified AGL and SRT as conceptually different, judgment-linked and motor-linked implicit learning, respectively. They appear to involve different cognitive processes. For example, while fluency does not play a role in AGL [46], speed-up revealed by RT benefits is the learning effect in the SRT task [17], [47]. Maybe global-local cultural differences are found in processes involving judgments but not in motor facilitation. Thus, it remains unclear that whether the cultural differences found in AGL would be reflected in the acquisition of unconscious knowledge in the SRT task.

The aim of the present study was to further investigate whether culture influences what people acquire in implicit sequence learning by adopting the global-local Navon letters in the SRT task, when participants are instructed to attend to one of the two levels. Previous studies have shown that an irrelevant sequence can be learnt, at least if it is limited to the first order transition probabilities [48], [49]. Given the previous findings that Asians process more background information [1], there may be cultural differences in the extent of processing of task irrelevant structures, as well as in the preferred global-local level. Thus, we adopted two types of regularity, at either the local or global level: a target regularity and a task irrelevant regularity. In the target regularity, target letters followed a second-order conditional (SOC) sequence. An SOC sequence means prediction requires knowing the two preceding letters. In the irrelevant regularity, irrelevant letters followed a first-order conditional (FOC) sequence. An FOC sequence means each letter can be predicted from the one preceding letter. The use of an SOC sequence for the attended level was to maximize the chance of obtaining implicit learning [15], [50]; the use of an FOC sequence for the unattended level was to maximize the chance of getting any learning at all [49]. As Eastern Asians rather than Westerners have biases for interdependence and are likely to have a holistic cognitive style, we predict Eastern Asians instead of Westerners acquire more knowledge of the regularity at the global level. Moreover, as Eastern Asians rather than Westerners are more sensitive to context [1], [3], we predict they instead of Westerners acquire more knowledge of the irrelevant regularity. This would be shown by reaction time differences between trials which obeyed or deviated from the regularity. Through comparing the reaction times for same global and local targets between normal people from different cultures early in learning, we can also directly examine whether there is a cultural difference in perception.

To test the conscious status of the acquired knowledge, two classification tests for the target and irrelevant regularities were used. On each test trial, participants were first asked to respond to several targets as in the training phase and then instructed to report whether the letter of the last target followed the FOC or SOC sequence. As unconscious knowledge may contribute to recognition performance, participants were asked to report the basis of their judgment of each test trial by indicating one of: guess, intuition, rules or memory (as used in various implicit learning paradigms by [20], [51]–[57]). Specifically participants indicate they are prima facie unaware of the basis of their judgments by saying that the judgment was made randomly (a *guess*), or it was based on feelings of *intuition*, and they have no idea why their judgment is right. Conversely, participants indicate they are prima facie aware of knowing the environmental regularity informing their judgment when they indicate it was based on *rules* they could state, or on *memory* of the sequence. That is, only when classification performance with a ‘rules or memory’ attribution is above chance is there evidence of the acquisition of conscious knowledge. See [58] for evidence that distinguishing conscious and unconscious structural knowledge by these attributions picks out different knowledge types, distinguished in ways expected on general theories of the difference between the conscious and unconscious.

## Methods

### Participants

Fifty-one Chinese undergraduate students (24 male, 27 female) and 51 British undergraduate students (26 male, 25 female) voluntarily took part in the experiment for payments or received course credit. Chinese and British were randomly assigned to the global and local target group (global-Chinese, *n* = 24; local-Chinese, *n* = 27; Global-British, *n* = 24; local-British, *n* = 27). Data from one British participant in the global group and three Chinese participants and two British participants in the local group were excluded because their error proportions were greater than .15.

### Ethics statement

The protocol used in this experiment was approved by the committee for the protection of subjects at the Institute of Psychology, Chinese Academy of Sciences and the School of Psychology Research Governance Committee, University of Sussex. Written consent for the collection of data and subsequent analysis was obtained from each participant.

### Apparatus and Materials

**Figure 1:**
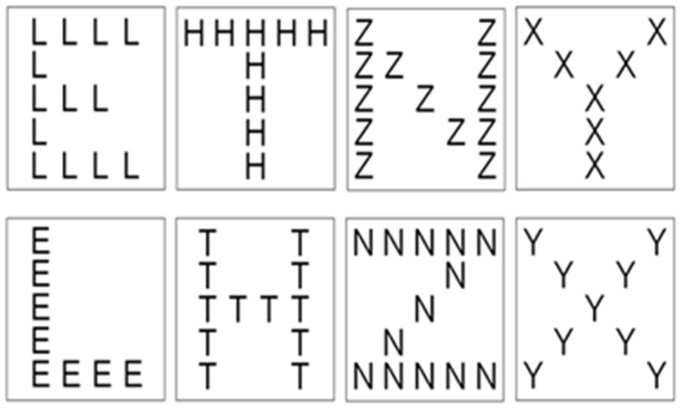
Stimulus examples for global and local target letters E, T, N, and Y with irrelevant letters H, L, X, and Z.

The experiment was programmed in E-prime 1.2. The display consisted of a stimulus in the centre of the computer's screen against a gray background. The stimuli were compound letters E, T, N, and Y, which were constructed out of or formed letters H, L, X, and Z (see Figure 1). Each letter could be either at global or local level. E, T, N, and Y were target letters while H, L, X, and Z were irrelevant letters. On each trial, a compound letter appeared in the square, which was in the center of the screen and covered a visual angle of approximately 2°.

### Procedure

#### Training phase

Participants were exposed to a serial four-choice RT task, which included one practice block and 12 training blocks. The practice block consisted of 98 trials, and each training block consisted of 98 trials, for a total of 1176 training trials. On each trial, a compound letter was presented and participants were instructed to discriminate target letters E, T, N, and Y as quickly and as accurately as possible by pressing the corresponding key. Keys D, F, J, and K corresponded to letters E, T, N and Y, respectively. Participants were required to press Keys D and F with the middle and index finger, respectively, of their left hand and to press Keys J and K with the index and middle finger, respectively, of their right hand. The letter was removed as soon as a correct key had been pressed, and the next stimulus appeared immediately. Response latencies were measured from the onset of the target to the completion of a correct response.

**Figure 2:**
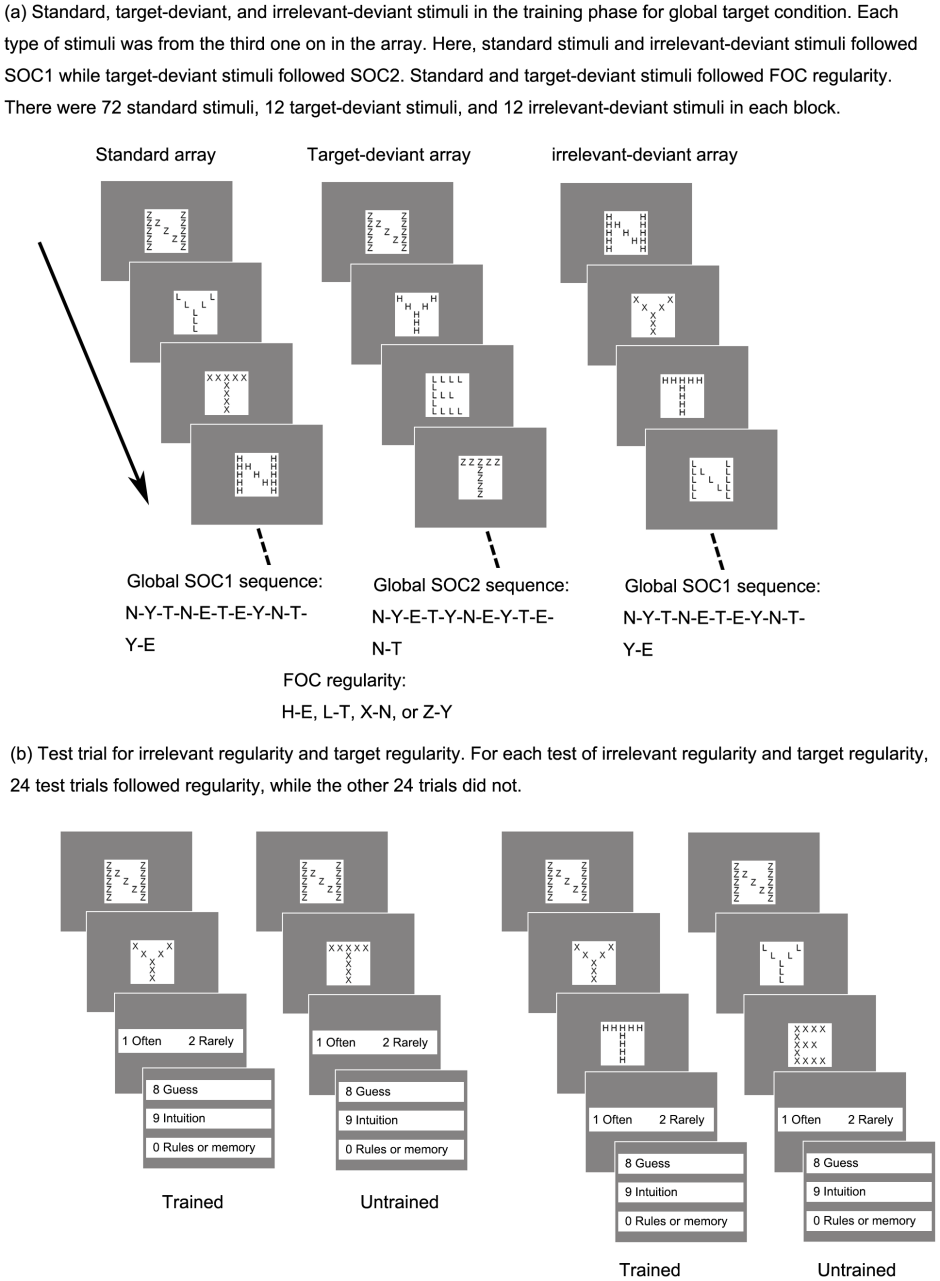
Experimental setup and design.

In the practice block, stimuli were randomly presented except that no two continuous stimuli were in the same target or irrelevant letters. In each training block, there were three types of stimuli: standard stimuli with a probability of .75, for which both target and irrelevant letters followed the regularity; target-deviant stimuli with a probability of .125, for which only irrelevant letters followed the regularity while target letters did not; irrelevant-deviant stimuli with a probability of .125, for which only target letters followed the regularity while irrelevant letters did not. For the target regularity, the target letters (E, T, N, and Y) were determined by the preceding two target letters. That is, the target letters followed one of two second-order conditional (SOC) sequences (SOC1 = N-Y-T-N-E-T-E-Y-N-T-Y-E; SOC2 = N-Y-E-T-Y-N-E-Y-T-E-N-T). For the irrelevant regularity, the target letters were determined by the irrelevant letter of the immediately preceding stimulus. That is, the relation of irrelevant letter and target letter followed a first-order conditional (FOC) sequence (H - E, L - T, X - N, or Z - Y). For example, on the basis of the SOC1 sequence, if the first two target letters were N and Y, the third target letter should be T; on the basis of the FOC sequence, if the first target letter N was made of the irrelevant letter Z, the second target letter should be Y (see Figure 2).

Each training block began at a random point in one of the two SOC sequences. It was continued by a sequence of 12 targets of one type of stimuli (i.e., standard or deviant), and then transferred to a sequence of 12 targets of another type of stimuli. Standard could transfer to any type of stimuli but deviant could transfer only to standard. For half of the participants in each group, standard targets followed the SOC1 and FOC regularity; for the other half, standard targets followed the SOC2 and FOC regularity. Target-deviant stimuli followed SOC2 or SOC1 depending on the SOC sequence standard stimuli followed and irrelevant-deviant stimuli changed with equal probability to one of the other three letters. The sequential positions of target-deviant and irrelevant-deviant stimuli in each block were counterbalanced during training. There were at least 30 seconds breaks between any two blocks.

#### Test phase

The test phase involved two classification tests: one for the target regularity, the other for the irrelevant regularity. At the beginning of each test, participants were informed that the target letters in the training had followed regularities. On each test trial for irrelevant regularity, participants were asked to first respond to two target letters as in the training, and then to report whether the second target letter followed the preceding irrelevant letter often (with a probability of about 90%) or rarely (with a probability of about 10%). On each test trial for target regularity, they were first asked to respond to three target letters as in the training, and then to report whether the third target letter followed the preceding two target letters often (with a probability of about 90%) or rarely (with a probability of about 10%). After each test trial in both tests, participants were required to report the basis of their judgment by ticking one of: guess, intuition, rules or memory; the definition of each of them was given as [51]. Specifically, participants were told that guess meant they responded randomly, they could just as well have flipped a coin, the judgment had no basis; intuition meant they had some confidence in their judgment but they had no idea why; rules meant they used a rule they could state if asked; and memory meant they remembered or failed to remember the sequence from the training phase. There were 48 test trials in each test, of which half followed the regularity and half did not. The irregular test trials were taken from target- or irrelevant-deviant sequence, respectively, except that the third target in the irregular test trials for target regularity could not be predicted by the irregular letter of the second one. Before each test, there were eight practice trials. Participants were first tested on the irrelevant regularity and then on the target regularity.

## Results

We will consider the following questions in order: Did culture influence initial perceptual preferences for a global or local scale? Then, crucially, did culture influence the learning of regularities at the global versus local level? Were people consciously aware of the learned regularity as shown by their discrimination ability on the classification test? Finally, we look at differences in response bias in the classification test.

### Did culture influence initial perceptual preference for a global or local scale?

Trials with RTs greater than 2,000 milliseconds were dropped; these amounted to 1.16%, 1.24%, 1.33% and 1.32% of the trials in Chinese and British global and local groups, respectively. Because there were only 12 deviant stimuli in each training block, the mean RTs were calculated for target-deviant, standard, irrelevant-deviant targets across pairs of successive blocks, giving six sessions in total.

**Figure 3:**
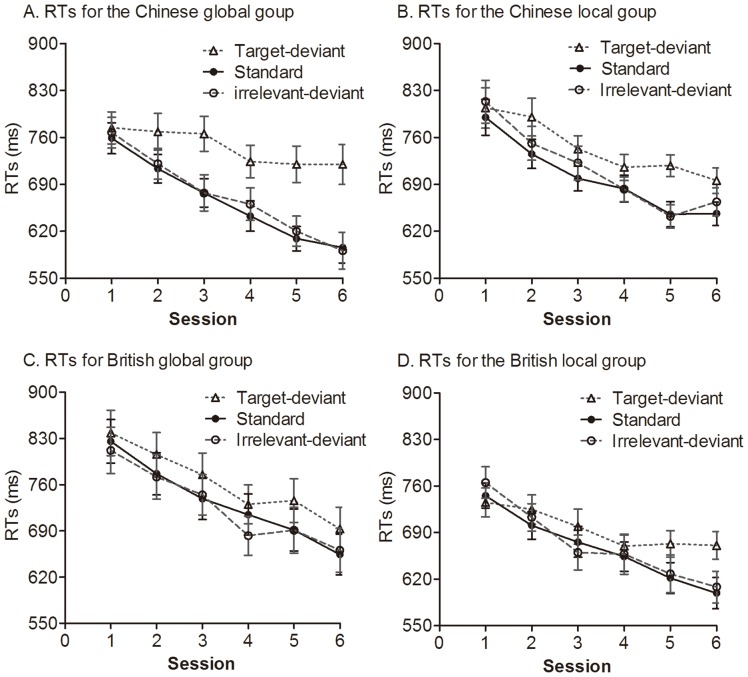
Mean reaction times (RTs) for target-deviant, standard, and irrelevant-deviant stimuli across training blocks in each group. Error bars depict standard errors.

**Figure 4:**
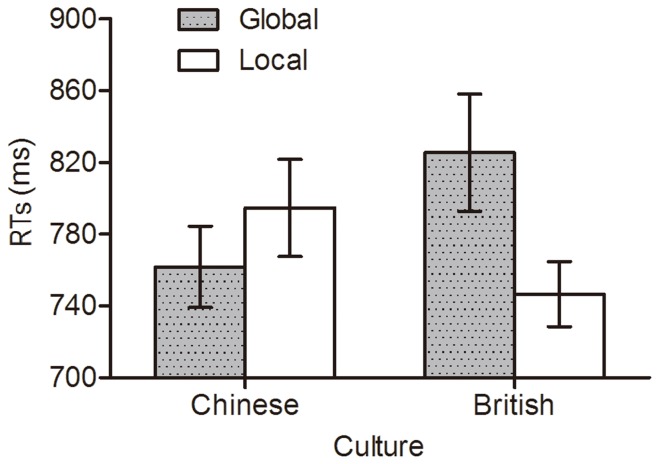
Mean reaction times (RTs) in session 1 in each group. Error bars depict standard errors.

Figure 3 shows mean RTs obtained over the training phase in each group. At the start of training, before learning has occurred, the RTs for global versus local targets reflect initial perceptual biases. An ANOVA on RTs in Session 1 with stimulus type (target-deviant vs. standard vs. irrelevant-deviant) as a within-subject variable, culture (Chinese vs. British) and target level (global vs. local) as between-subjects variables revealed only a significant target level by group interaction, *F* (1, 92) = 4.83, *p*<.05, η*_p_* ^2^ = .05 (see Figure 4). British participants responded to local target letters faster than to global target letters, *t* (46) = 2.15, *p*<.05, *d* = .62; Chinese participants responded to global target letters non-significantly faster than local target letters, *t* (46) = −.93, *p* = .36. That is, British more than Chinese people initially expressed a local bias in perception.

### Did culture influence learning?

**Figure 5:**
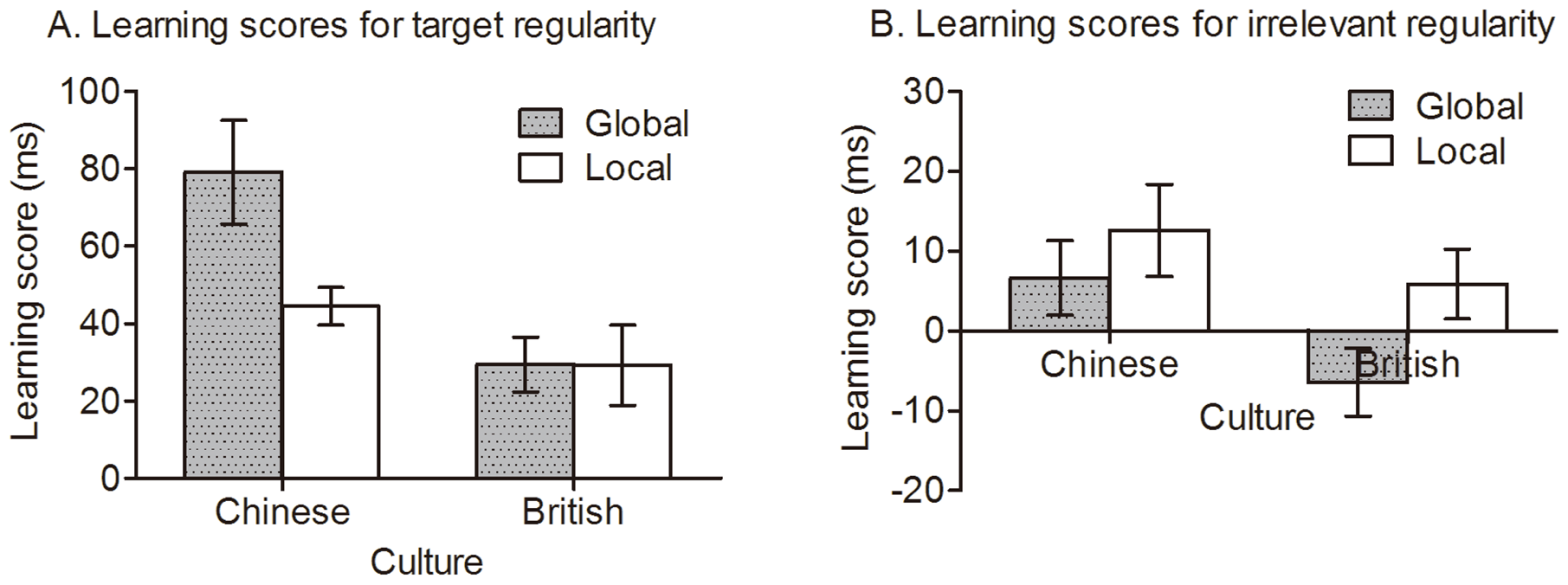
Learning scores of target and irrelevant regularities in each group. Error bars depict standard errors.

If participants learned the target regularity, they would respond to the standard letters faster than to the target-deviant targets; similarly, if participants learned the task-irrelevant regularity, they would respond to the standard targets faster than the irrelevant-deviant targets. Thus, the RT differences between standard and deviant targets were taken as a measure of learning. To examine how learning was influenced by culture and target level, a learning score was calculated separately for the target and task-irrelevant regularities. The learning score for the target regularity was calculated as the RT to target-deviant letters minus the RT to standard letters, while the learning score for the task-irrelevant regularity was calculated as the RT to irrelevant regularity minus the RT to standards. If there were no learning, this score would be zero; if there were learning, the score would be above zero. Figure 5 shows these summarized data.

We report separate analyses for target and irrelevant regularities. One -sample *t* tests were used to compare learning scores with chance (i.e. zero) for each group. This revealed that the target regularity was learned significantly by all groups (all *p*s<.001), but the task irrelevant regularity was learned significantly only by the Chinese-local group in which the irrelevant letters were global, *t* (23) = 2.19, *p*<.05, *d* = .46. To test whether culture influenced the learning of target regularity, an ANOVA on learning scores of target regularity with culture (Chinese vs. British) and target level (global vs. local) as between-subjects variables was used. It revealed a significant culture effect, *F* (1, 92) = 11.50, *p*<.001, η*_p_* ^2^ = .11, which was modulated by a marginally significant culture by target level interaction, *F* (1, 92) = 3.24, *p* = .075, η*_p_* ^2^ = .03. Chinese participants learned the target regularity significantly better than the British when the target letters were at global level, *t* (45) = 3.24, *p*<.01, *d* = .95, but not when they were at the local level, *t* (47) = 1.31, *p* = .20. Moreover, Chinese participants learned the target regularity better for global than for local target letters, *t* (46) = 2.43, *p*<.05, *d* = .70, but British participants learned the global and local target regularities similarly, *t* (46) = .02, *p* = .99. In sum, Chinese rather than British people had a greater global advantage in learning the target regularity.

To test whether culture influence the learning of irrelevant regularity, a comparable ANOVA on learning scores of the irrelevant regularity was used. It revealed only a culture effect, *F* (1, 92) = 4.24, *p*<.05, η*_p_* ^2^ = .044, indicating that Chinese participants acquired the irrelevant regularity better than the British participants. Specifically, the Chinese local group expressed significant knowledge of the irrelevant regularity when irrelevant letters were global, *t* (23) = 2.19, *p*<.05, *d* = .46, but not when irrelevant letters were local, *t* (23) = 1.43, *p* = .17. However, the difference between global and local targets was non-significant, *t* (46) = .81, *p* = .42, so any possible global advantage of Chinese for unattended stimuli is neither supported nor refuted. There was no significant learning for British participants in either group, *t* (22) = −1.51, *p* = .15, *t* (24) = 1.34, *p* = .19, respectively. Thus, overall, the results indicate an advantage for Chinese over British in learning irrelevant structures.

The error proportions were .08, .05, .05 and .06, .04, .04 for target-deviant, standard, irrelevant-deviant stimuli in Chinese global and local groups, respectively, and .10, .07, .07 and .09, .06, .06 for target-deviant, standard, irrelevant-deviant stimuli in British global and local groups, respectively. None of the significant RT effects were compromised by possible speed-error trade-offs.

A mixed ANOVA on error rates with culture (Chinese vs. British) and target level (global vs. local) as between-subjects variables and type of stimuli (target-deviant vs. standard vs. irrelevant-deviant) as a within-subject variable revealed only a significant type of stimuli effect, *F* (2, 184) = 64.01, *p*<.001, η*_p_* ^2^ = .41, and a significant culture effect, *F* (1, 92) = 9.25, *p*<.01, η*_p_* ^2^ = .09. The interaction of culture by type of stimuli on error rates was not significant, *F* (2, 184) = .09, *p* = .91. The error rates for standard were smaller than those for target-deviant stimuli in both British and Chinese groups (*M* = .03, *p*<.001, *M* = .03, *p*<.001, respectively) and there was no significant difference between standard and irrelevant-deviant stimuli in either group (*M* = .001, *p* = .74, *M* = .000, *p* = .89).

### Were people consciously aware of the irrelevant and target regularities?

RT differences can indicate only whether a regularity was acquired, not its conscious status. Thus, the recognition tests were used to further assess whether the regularity was consciously learned. If participants consciously learned the regularity, they would classify correctly the test trial as from the training or deviant sequence. Conversely, if population accuracy were at chance, the acquired knowledge would be unconscious. We used *A*′ (similar to *d*′) as an estimate of sensitivity of discrimination, which is used widely in recognition tests to control response bias [59], [60]. To compute *A*′, we first quantified the hit rate (H) as the proportion of correct responses for test trials from the training sequence and the false-alarm rate (F) as the proportion of incorrect responses for test trials from the deviant sequence. *A*′ was calculated as follows: *A*′ = 0.50+(H−F) (1+H−F)/[4H (1−F)], when H≥F, and *A*′ = 0.50−(F−H) (1+F−H)/[4F (1−H)], when H<F ([59], equation 2). *A*′ ranges from 0 to 1, in which 0.50 indicates that signals cannot be distinguished from noise, 1 corresponds to perfect performance, and values less than 0.50 indicates discrimination in the wrong direction (a particularly strong indicator of unconscious knowledge according to [61]). We calculated the *A*′ both overall and for each attribution.

Table 1 shows mean proportions for each attribution of each group in the classification test for irrelevant regularity. An ANOVA on proportions with attribution (guess vs. intuition vs. rules or memory) as a within-subject variable and culture (Chinese vs. British) and target level (global vs. local) as between-subjects variables was used. It revealed only an attribution effect, *F* (2, 184) = 10.69, *p*<.001, η*_p_* ^2^ = .10. There were more intuition and rules or memory than guess attributions, *t* (95) = 4.25, *p*<.001, *dz* = .44, *t* (95) = 4.42, *p*<.001, *dz* = .45, respectively, and no significant difference between intuition and rules or memory attributions, *t* (95) = 1.09, *p* = .28. Moreover, overall, participants gave more unconscious (guess and intuition) judgments than conscious judgments (rules or memory), *t* (95) = 2.37, *p*<.05, *dz* = .24.

**Table 1:** Mean Proportions for Each Attribution of Each Group in the Classification Test for Irrelevant Regularity.

		G	I	R or M
Global	Chinese	.20 (.05)	.34 (.06)	.45 (.08)
	British	.22 (.03)	.40 (.05)	.38 (.06)
Local	Chinese	.19 (.05)	.38 (.06)	.42 (.07)
	British	.23 (.04)	.34 (.04)	.44 (.05)

Note: G, I, and R or M refer to the trials in which participants gave guess, intuition, and rules or memory attribution, respectively.

Standard Errors in Brackets.

Table 2 shows mean values of *A*′ for each attribution of each group in the classification test for irrelevant regularity. Even though *A*′ is bounded, means were close to baseline, and stem and leaf plots indicated that the distributions were roughly normal. Because only Chinese participants in the local group acquired significant knowledge about the task-irrelevant regularity, while overall, Chinese participants learned the irrelevant regularity better than British, we compared the performance of just Chinese groups with the baseline (0.50). One-sample *t*-test revealed that Chinese participants in the local and global groups performed non-significantly different from chance, *t* (22) = −.64, *p* = .53, *t* (22) = .64, *p* = .53, respectively.

**Table 2:** Mean Values of A′ for Each Attribution of Each Group in the Classification Test for Irrelevant Regularity.

		Total	G	I	R or M
Global	Chinese	.52 (.03)	.51 (.07)	.54 (.05)	.53 (.04)
	British	.56 (.03)	.68 (.05)	.55 (.04)	.53 (.06)
Local	Chinese	.48 (.03)	.35 (.07)	.51 (.04)	.49 (.06)
	British	.50 (.02)	.59 (.07)	.45 (.05)	.51 (.05)

Note: G, I, and R or M refer to the trials in which participants gave guess, intuition, and rules or memory attribution, respectively.

Standard Errors in Brackets.

In order to interpret this null result, we first estimated what the expected recognition performance would be if the knowledge were all conscious. The irrelevant-regularity included four rules: H - E, L - T, X - N, and Z – Y. We calculated and compared the RTs for standard and irrelevant-deviant targets for each of the four rules through the training. For Chinese participants in the local group, three rules were learned significantly (*t* (23) = 2.78, *p*<.05, *d* = .58, *t* (23) = 2.98, *p*<.01, *d* = .62, *t* (23) = 3.70, *p* = .001, *d* = .77, respectively), while one was learned the wrong way (*t* (23) = −3.75, *p* = .001, *d* = .78). If this knowledge were completely conscious, people would recognize correctly the three rules and recognize incorrectly one, producing an expected hit performance (1*3+0*1)/4 = .75 and an expected false alarm performance (0*3+1*1)/4 = .25, i.e. the *A*′ would be .83. For Chinese participants in the global group, one rule was learned significantly (*t* (23) = 3.72, *p* = .001, *d* = .78), while three rules were not learned (all *p*s>.10). Similarly, if this knowledge were completely conscious, people would recognize correctly the one rule and recognize the other three at chance, producing an expected hit performance (1*1+0.5*3)/4 = .625 and an expected false alarm performance (1*0+0.5*3)/4 = .375, i.e. the *A*′ would be .70. The upper limit of the confidence interval on recognition performance was .54 and .57 for local and global groups separately, substantially below the expected performance, consistent with the knowledge about the irrelevant regularity being unconscious or implicit. However, for the Chinese local group, .83 defines the upper limit expected on the theory that even all knowledge is conscious because there could be random noise in any given recognition judgment, for example, as postulated by [62]. Given this noise could be any amount from 0% to 100% of the recognition signal, the expected recognition performance could be any value from .50 to .83 with equal probability. This hypothesis can be compared against the null that all knowledge was unconscious with a Bayes factor. Bayes Factors vary between 0 and infinity with values of less than .33 indicating support for the null hypothesis and values greater than 3 indicating support for the alternative. Values in between indicate data insensitivity (see [63], [64], for explanation of Bayes Factors and free online software). The Bayes Factor was .18 for the Chinese local group, strong evidence against the theory of partial or complete conscious knowledge and in favor of the null hypothesis of all knowledge being unconscious. Similarly, for the Chinese global group, the Bayes Factor was .20, also providing strong evidence in favor of the null hypothesis of no conscious knowledge.

After each test trial, participants reported the basis (i.e., guess, intuition, rules or memory) of their judgment. However, because of the overall chance-level performance we did not further analyze the accuracy rates for each attribution.

Table 3 shows mean proportions for each attribution of each group in the classification test for target regularity. An ANOVA on proportions with attribution (guess vs. intuition vs. rules or memory) as a within-subject variable and culture (Chinese vs. British) and target level (global vs. local) as between-subjects variables was used. It revealed only an attribution effect, *F* (2, 184) = 8.28, *p*<.001, η*_p_* ^2^ = .08. As for the FOC test, there were more intuition and rules or memory than guess attributions, *t* (95) = 3.66, *p*<.001, *dz* = .38, *t* (95) = 3.74, *p*<.001, *dz* = .38, respectively; there was no significant difference between intuition and rules or memory attributions, *t* (95) = .49, *p* = .62. Moreover, overall, participants gave more unconscious (guess and intuition) judgments than conscious judgments (rules or memory), *t* (95) = 3.24, *p*<.01, *dz* = .33.

**Table 3:** Mean Proportions for Each Attribution of Each Group in the Classification Test for Target Regularity.

		G	I	R or M
Global	Chinese	.24 (.05)	.29 (.04)	.47 (.07)
	British	.20 (.03)	.43 (.05)	.37 (.06)
Local	Chinese	.22 (.06)	.40 (.06)	.38 (.06)
	British	.23 (.05)	.39 (.06)	.39 (.05)

Note: G, I, and R or M refer to the trials in which participants gave guess, intuition, and rules or memory attribution, respectively.

Standard Errors in Brackets.

Table 4 shows mean values of *A*′ for each attribution of each group in the classification test for the target regularity. One-sample *t*-test revealed that British participants performed at chance level for the local target letters, *t* (21) = −.67, *p* = .51, but, surprisingly, significantly below chance level for the global target letters, *t* (22) = −2.47, *p*<.05, *d* = .54. Further analysis of *A*′ for each attribution revealed that conscious judgment was not significantly above baseline for either British global or local groups (*p*s>.09). Chinese participants performed at chance for local target letters, *t* (21) = .96, *p* = .35, but marginally above chance for global target letters, *t* (22) = 1.87, *p* = .075 (*M* = .56, *SE* = .03). Further analysis revealed that only Chinese participants in the global group performed significantly above chance for rules or memory attributions, *t* (19) = 2.20, *p*<.05, *d* = .50, but not in the local group, *t* (21) = .96, *p* = .35 (*M* = .53, *SE* = .03), indicating that the global Chinese group were at least partially consciously aware of the global target regularity.

**Table 4:** Mean Values of A′ for Each Attribution of Each Group in the Classification Test for Target Regularity.

		Total	G	I	R or M
Global	Chinese	.56 (.03)	.43 (.06)	.56 (.05)	.60 (.05)
	British	.44 (.02)	.52 (.05)	.43 (.04)	.41 (.05)
Local	Chinese	.53 (.03)	.49 (.09)	.56 (.05)	.55 (.06)
	British	.48 (.03)	.41 (.07)	.44 (.05)	.49 (.05)

Notes: G, I, and R or M refer to the trials in which participants gave guess, intuition, and rules or memory attribution, respectively.

Standard Errors in Brackets.

To determine how much recognition performance would be expected if the knowledge acquired in the training were conscious, we calculated and compared the RTs for standard and target-deviant for each of the twelve different triplets in the training sequence in each group. For Chinese participants in the global group, ten triplets were learned significantly (all *t*s>1.92) while two were not (both *p*s>.17). Thus, if this knowledge were conscious, people should recognize ten triplets correctly, and guess correctly half of the remaining two triplets. Thus, people's expected hit performance on the recognition test would be (1*10+0.5*2)/12 = .92, and false alarm performance would be (0*10+0.5*2)/12 = .08, so the *A*′ would be .95. Similarly, for the Chinese local group, five triplets were learned significantly (all *t*s>1.73) while seven were not (all *p*s>.18). People's expected hit performance on the recognition test would be (1*5+0.5*7)/12 = .71, and false alarm performance would be (0*5+0.5*7)/12 = .29, so the *A*′ would be .79. In fact the upper limit of the confidence interval on *A*′ was .62 and .59 for the Chinese global and local groups, substantially below the expected performance on the hypothesis of completely conscious knowledge. As before, the Bayes Factors pitting the theory that classification performance could be any value from .50 to .95 or .79 with equal probability against the null were .95 and .35 for the Chinese global and local groups, respectively, indicating little sensitivity to distinguish the theories, especially in the former case.

However, the Bayes Factors assume that after 12 blocks of training there could still be as high as 100% noise in classification even though the knowledge was all conscious. [62] argues that in fact the noise in recognition is small compared to priming effects. If we assume the noise is even as high as 90% to allow hit performance as low as 0.5+(0.92−0.5) * (1−.90) = .54 and 0.5+0.71−0.5) * (1−.90) = .52 (i.e., A′ = .58, A′ = .54), so we can represent the theory of complete conscious knowledge as a uniform between .58 and .92 or between.54 and .79, the Bayes Factor are .29 and .16 for Chinese global and local groups separately, providing evidence in favor of the null hypothesis. This conclusion of course is conditional on accepting 90% noise as a maximum. In fact, we know from the more refined attribution judgments that in the global condition there was a small amount of conscious knowledge for Chinese.

For the British groups, participants in each group learned four triplets significantly (all *t*s>1.78) while eight were not (all *p*s>.11) for the British global and local groups. The people's expected hit performance on the recognition test would be (1*4+0.5*8)/12 = .67, and false alarm performance would be (0*4+0.5*8)/12 = .33, so the *A*′ would be .75, which were also substantially above the upper limit of the confidence interval of .49 and .54. As before, the Bayes Factors pitting the theory that recognition performance could be any value from .50 to .75 with equal probability against the null were .04 and .08 for British global and local groups, respectively, providing strong evidence in favor of the null hypothesis that all knowledge was unconscious and against that the theory of partial or complete conscious knowledge.

### Was there a cultural difference in response bias in classification?

We used B″ (similar to *β*) as an estimate of response bias [59]. *B*″ was calculated as follows: *B*″ = [H (1−H)−F (1−F)]/[H (1−H)+F (1−F)], when H≥F, and *B*″ = [F (1−F)−H (1−H)]/[F (1−F)+H (1−H)], when H<F ([59], equation 8). *B*″ ranges from −1 to 1, in which −1 means extreme bias in favor of *yes* response, 1 means extreme bias in favor of *no* response, and 0 means no response bias. Tables 5 shows mean values of *B*″ for each attribution of each group in the classification test for irrelevant regularity. An ANOVA on *B*″ with culture (Chinese vs. British) and target (global vs. local) as between-subjects variables revealed no significant effects (all *p*s>.11). Further, an ANOVA on *B*″ with attribution (guess vs. intuition vs. rules or memory) as a within-subject variable and culture (Chinese vs. British) and target (global vs. local) as between-subjects variables revealed only an attribution effect, *F* (2, 74) = 3.69, *p*<.05, η*_p_* ^2^ = .09. People gave more *no* responses when attributing to guess and intuition than rules or memory, *t* (40) = 2.67, *p*<.05, *dz* = .42, *t* (40) = 2.60, *p*<.05, *dz* = .41, respectively; there was no significant difference between guess and intuition attributions, *t* (40) = .77, *p* = .44. That is, participants gave more *no* responses for unconscious judgment than for conscious judgment. In addition, one sample *t* test revealed that people expressed a significant bias in favor of *yes* responses when attributing to rules or memory, *t* (72) = −3.79, *p*<.001, *dz* = −.45, but did not when attributing to guess and intuition (both *p*s>.37).

**Table 5:** Mean Values of B″ for Each Attribution of Each Group in the Classification Test for Irrelevant Regularity.

		Total	G	I	R or M
Global	Chinese	.04 (.05)	.18 (.17)	.08 (.08)	−.13 (.07)
	British	−.04 (.01)	.10 (.14)	.01 (.07)	−.50 (.09)
Local	Chinese	.03 (.06)	.04 (.22)	.13 (.08)	−.07 (.16)
	British	−.01 (.03)	−.02 (.14)	−.05 (.12)	−.08 (.10)

Note: G, I, and R or M refer to the trials in which participants gave guess, intuition, and rules or memory attribution, respectively.

Standard Errors in Brackets.

Tables 6 shows mean values of *B*″ for each attribution of each group in the classification test for the target regularity. An ANOVA on *B*″ with culture (Chinese vs. British) and target (global vs. local) as between-subjects variables revealed a significant culture effect, *F* (1, 86) = 6.58, *p*<.05, η*_p_* ^2^ = .07, indicating that Chinese people gave more *no* responses than British people. Indeed, Chinese people also tended to give more no responses (*M* = .04, *SE* = .03) than British people (*M* = −.03, *SE* = .03) in the FOC test, although it did not reach significance, *t* (90) = 1.61, *p* = .11. Further, an ANOVA on *B*″ with attribution (guess vs. intuition vs. rules or memory) as a within-subject variable and culture (Chinese vs. British) and target (global vs. local) as between-subjects variables revealed only an attribution effect, *F* (2, 84) = 10.68, *p*<.001, η*_p_* ^2^ = .20. People gave more *no* responses when attributing to guess and intuition than rules or memory, *t* (45) = 3.94, *p*<.001, *d* = .59, *t* (45) = 2.82, *p*<.01, *d* = .42, respectively; there was no significant difference between guess and intuition attributions, *t* (45) = 1.73, *p* = .09, confirming that participants would give more *no* responses for unconscious judgment than conscious judgment. Finally, one sample *t* test revealed that people expressed a bias in favor of *no* responses when attributing to guess, *t* (56) = 2.17, *p*<.05, *dz* = .29, but a bias in favor of *yes* responses when attributing to rules or memory, *t* (74) = 4.07, *p*<.05, *dz* = .47, and no significant bias when attributing to intuition, *t* (74) = −.08, *p* = .94.

**Table 6:** Mean Values of B″ for Each Attribution of Each Group in the Classification Test for Target Regularity.

		Total	G	I	R or M
Global	Chinese	.08 (.05)	.23 (.11)	.15 (.11)	−.07 (.10)
	British	−.05 (.02)	.12 (.11)	−.12 (.07)	−.22 (.12)
Local	Chinese	.00 (.03)	.27 (.18)	.03 (.11)	−.43 (.12)
	British	−.05 (.03)	.00 (.15)	−.06 (.12)	−.21 (.09)

Note: G, I, and R or M refer to the trials in which participants gave guess, intuition, and rules or memory attribution, respectively.

Standard Errors in Brackets.

## Discussion

The results showed that British participants expressed a greater local bias in perception then Chinese participants, confirming the established cultural differences in conscious perception between Westerners and Asians [3]. Importantly, the Chinese rather than the British showed a stronger global advantage in implicit sequence learning. Moreover, Chinese participants acquired more unconscious knowledge of the irrelevant regularity than British participants (perhaps especially when the irrelevant letters were global), indicating that the Chinese were more sensitive to contextual regularities than the British. The findings are conceptually consistent with previous research [5], [35] and extend such research by demonstrating for the first time a global advantage for Chinese rather than Westerners in implicit sequence learning.

Previous research [35]–[37], using a self-construal priming paradigm, found a global precedence in an interdependent priming condition but a local precedence in an independent priming condition using Navon letters. These findings suggest cross cultural differences should be found in perceiving Navon letters, given Asians value inter-dependence and Westerners value independence [3]. Consistently, through directly comparing the RTs to global or local target letters between Chinese and British people, we found that British participants expressed a greater local bias than Chinese, confirming a cultural difference in perception. Importantly, in terms of the main aim of the paper, our results show for the first time that Chinese participants learned more knowledge of a target regularity when the target letters were global than when the target were local, and Chinese participants performed better than British participants when the target letters were global, i.e., expressed a global advantage in implicit sequence learning. This finding is consistent with [42], who found that there was a greater global advantage of Japanese rather than British in implicit artificial grammar learning. The joint findings show that cultural biases can not only influence what people consciously perceive but also profoundly influence what people unconsciously learn.

Whereas [42] found a global-local difference between Asians and Westerners in implicit learning when they were left to attend to the level that they preferred, we found a difference even when participants were told to which level they should attend. Thus, the different content of unconscious knowledge acquired by Asians and Westerners relies as much on skill differences as habit differences (cf.[43]). [42] used what Seger [45] called a judgment-linked implicit learning task, i.e., knowledge was expressed by what judgments people made, whereas we used what Seger [45] called a motor-linked implicit learning task, i.e., knowledge was expressed in the speed of motor response, not in the content of judgments. While both sorts of tasks are similar in many ways and thus can be modeled by the same types of computational model (e.g.[65]), they also have different properties, for example in whether the knowledge is expressed as fluency [46], [66]. Both types of implicit learning task have now been shown to be sensitive to cross cultural differences in preferences to attend to global versus local levels. Whether judgment-linked tasks can be shown sensitive to skill rather than just habit difference between the cultures has not yet been shown.

Moreover, we also found that when paying attention to the target letters, Chinese participants acquired greater knowledge about the irrelevant regularity than British participants, indicating that Chinese people were more sensitive to the contextual regularities than British people. However, [42] found no evidence that either Japanese or British participants could acquire the grammar of the unattended level in the artificial grammar learning task. This might because that the irrelevant (i.e., unattended) letters in the SRT were useful cues to the target (i.e., attended) letters, while the irrelevant letters in the AGL had nothing to do with the target letters. Thus, the irrelevant sequence was only partially irrelevant in our case, whereas it was completely irrelevant in [42]. Consistent with the role of relevance postulated by [25], task relevance may have facilitated learning at the unattended level (cf also [67]). However, as [42] did not analyze the null result with Bayes Factors, the null hypothesis of no learning cannot yet be asserted for their paradigm.

British participants made more errors overall than Chinese participants. Thus, the different learning effects between British and Chinese participants might be because British participants were less motivated to take the task seriously. However, if this were the case, Chinese participants would learn the target regularity better than British participants at the local level to the same extent as they do at the global level. In fact, there was no difference between British and Chinese participants at the local level, *t* (47) = 1.31, *p* = .20; a Bayes factor of .26 indicating that this non-significant result is strong evidence for the null hypothesis (prediction of the theory that motivation explains cultural differences modeled as a normal with mean equal to the difference between cultures at the global level (i.e. 50) and an SD equal to the SD of the sampling distribution of that estimate (i.e. 14)). Further, the cultural group by target level interaction (*p*<.05 1-tailed) also indicated a selective superiority of the Chinese over the British at the global level. Therefore, motivation cannot be the main reason for Chinese performing better than British.

We established that the knowledge was largely unconscious because of the poor performance of participants in classifying standard and deviant sequences. Claims that knowledge on the SRT task is unconscious because performance on a recognition or classification test is at chance are common (see [8]–[9], [68]). However, in itself this methodology is insufficient. Given that even complete conscious knowledge may express itself only partially on a given set of trials (e.g., [62]), recognition or classification performance may be anything from chance upwards even for completely conscious knowledge. This possibility renders the theory that subjects have completely conscious knowledge expressed partially (or partial conscious knowledge expressed completely) unfalsifiable using significance or hypothesis testing on recognition performance. Conversely, the theory that all the knowledge is unconscious cannot be empirically distinguished from the claim the test was insensitive, using significance or hypothesis testing on recognition or classification performance. So the way in which researchers have been using the method of recognition or classification tests with significance testing, the claim for unconscious knowledge based on non-significant performance has not had coherent foundations. Bayes Factors offer the only solution, because they express the strength of evidence for one theory over another, something that significance testing does not allow [69].

In order to calculate a Bayes Factor, the rough typical or maximum score allowed by the theory should be specified. We followed a procedure introduced by [70] of using the RT data to determine evidence for how much of the structure was learnt in the SRT task. We improved the technique in one way; whereas [70] used percent correct recognition as the dependent variable we used A′ (a variant of d′) to take into account response bias. We showed that in most cases the poor performance was not simply a matter of test insensitivity; rather Bayes factors showed that, for most cases, the evidence positively supported the null hypothesis that all knowledge was unconscious, by taking into account the amount of knowledge expressed on the RT task. The one exception was for the global Chinese group, who performed above chance when they gave rules or memory attributions for the target regularity, indicating they acquired some conscious knowledge. However, the performance was considerably below the performance which would be expected if all acquired knowledge could be intentionally consciously retrieved. Thus, the knowledge the global Chinese group acquired was plausibly mainly unconscious. The British participants on both global and local target regularities and Chinese participants on the local target regularity appeared to acquire completely unconscious knowledge. In fact, the British participants trained on the global target regularity were discriminating at significantly below chance levels. It is not clear why this is, and is perhaps should be treated as a Type I error. We have no theory for why this task should produce such a finding. Nonetheless, [61] argue that below chance discrimination is particularly compelling evidence for knowledge being unconscious.

Our experimental procedure could be used to explore the relevance of global and local processing in implicit learning quite beyond the issue of cultural differences. For example, [70] and [71] argued that happy versus sad moods promoted the implicit learning of structures over greater time windows. It may also be that happy versus sad moods promote the implicit learning of more global rather than local structures considered spatially.

In sum, our findings suggest that cultural biases can profoundly influence both what people consciously perceive and unconsciously learn. Chinese people rather than British people were more sensitive to the contextual regularity and expressed a global advantage in the acquisition of both target and task-irrelevant regularities.
